# Desalination at ambient temperature and pressure by a novel class of biporous anisotropic membrane

**DOI:** 10.1038/s41598-022-17876-8

**Published:** 2022-08-09

**Authors:** Mohammed Rasool Qtaishat, Mohammed Obaid, Takeshi Matsuura, Areej Al-Samhouri, Jung-Gil Lee, Sofiane Soukane, Noreddine Ghaffour

**Affiliations:** 1 Chemical Engineering Department, School of Engineering, The University of Jordan, Amman, 11942 Jordan; 2 Arab Open University/ Jordan Branch, Amman, 11731 Jordan; 3 Saudi Membrane Distillation Desalination (SMDD) Co. Ltd., Innovation and Economic Development, King Abdullah University of Science and Technology (KAUST), Thuwal, 23955-6900 Saudi Arabia; 4 Division of Biological and Environmental Science and Engineering (BESE), Water Desalination and Reuse Center (WDRC), King Abdullah University of Science and Technology (KAUST), Thuwal, 23955-6900 Saudi Arabia; 5 Chemical and Biological Engineering Department, University of Ottawa, 161 Luis Pasteur Street, Ottawa, ON K1N 6N5 Canada; 6 Carbon Neutral Technology R&D Department, Korea Institute of Industrial Technology, 89, Yangdaegiro-gil, Seobuk-gu, Cheonan-si, Chungcheongnam-do 31056 South Korea

**Keywords:** Chemical engineering, Organic-inorganic nanostructures

## Abstract

Recent scientific advances have made headway in addressing pertinient issues in climate change and the sustainability of our natural environment. This study makes use of a novel approach to desalination that is environment friendly, naturally sustainable and energy efficient, meaning that it is also cost efficient. Evaporation is a key phenomenon in the natural environment and used in many industrial applications including desalination. For a liquid droplet, the vapor pressure changes due to the curved liquid–vapor interface at the droplet surface. The vapor pressure at a convex surface in a pore is, therefore, higher than that at a flat surface due to the capillary effect, and this effect is enhanced as the pore radius decreases. This concept inspired us to design a novel biporous anisotropic membrane for membrane distillation (MD), which enables to desalinate water at ambient temperature and pressure by applying only a small transmembrane temperature gradient. The novel membrane is described as a super-hydrophobic nano-porous/micro-porous composite membrane. A laboratory-made membrane with specifications determined by the theoretical model was prepared for model validation and tested for desalination at different feed inlet temperatures by direct contact MD. A water vapor flux as high as 39.94 ± 8.3 L m^−2^ h^−1^ was achieved by the novel membrane at low feed temperature (25 °C, permeate temperature = 20 °C), while the commercial PTFE membrane, which is widely used in MD research, had zero flux under the same operating conditions. As well, the fluxes of the fabricated membrane were much higher than the commercial membrane at various inlet feed temperatures.

## Introduction

One of the main points of contention nowadays revolves around energy consumption and its effect on our natural environment, especially in terms of the release of large amounts of carbon dioxide and the detrimental impact of this on global warming. Based on an essential belief in the sustainability of our natural environment combined with our knowledge of the behaviour of water vapor transport according to the kelvin equation, this research is being proposed as a breakthrough study in desalination technology. The technology being advanced here promises to resolve many problems that lower income countries are currently confronting in terms of the high cost of energy and the disastrous effect energy consumption has on the seemingly unstoppable process of climate change.

Desalination is a general term used for the methods that produce fresh water from salty water. The current desalination technologies are energy intensive since they require application of significant thermal or pressure driving force. Luckily, the thermal demand of evaporation in membrane processes such as membrane distillation (MD) and pervaporation is lower than in traditional distillation processes. Thus, MD is a thermal-driven separation technology that can potentially use low-grade heat to desalinate highly saline streams. In MD, driven by the partial pressure gradient across a hydrophobic microporous membrane, water vapor molecules transfer from the hot saline feed to the cold permeate, leaving salts and non-volatiles behind^1,2^. Keeping the membrane hydrophobic is crucial in MD because it enables the high salt rejection by preventing the salty feed water from flowing through the membrane pores into the permeate side (water product)^3,4^. MD has drawn much attention recently as an emerging desalination technology, due to its excellent features, such as low operating temperature, low operating pressure, high capability to treat high-salinity brines, high rejection efficiency, and unique ability for using low-grade energy sources^3,5^.

As mentioned earlier, MD process relies mainly on evaporation that consumes thermal energy. However, heat is also lost via conduction through the membrane, thereby decreasing the overall efficiency of the MD process, especially in DCMD configuration^6^. Thus, the fabrication of poorly designed membranes is often the reason for the high energy consumption as well as the decline of the MD performance^7^. The MD membranes ideally designed for high performance should fulfill the following requirements, i.e., low resistance for vapor transfer, small thickness, low thermal conductivity, high hydrophobicity, and excellent mechanical stability and durability^8,9^. Awareness of the coupling, and sometimes conflicting, influences of many parameters is crucial in designing high-performance MD membranes. Thus, all the above criteria need to be taken into consideration simultaneously to develop an effective MD membrane. For example, although the thinner MD membrane is expected to decrease the mass transfer resistance, a small thickness often exhibits low mechanical properties and increases conductive heat losses, especially in the case of DCMD^10^. Therefore, many theoretical studies have been conducted not only for understanding the effects of the parameters but also for optimizing them to achieve the highest possible MD performance^11,12^.

Despite the large amount of efforts, both theoretical and experimental, to improve MD performance, its intrinsic limitations have, so far, not allowed to maintain a high thermal driving force across the membrane, especially when a large membrane area is used. Thus, novel approaches to increase the thermal driving force as much as possible are highly demanded, especially to bring MD to the next level of large scale commercialization.

The objective of this work is to present one of the methods to fulfill such requirements based on the fundamental concept of vapor pressure increase due to the capillary effect in nano-sized pores^13^. A theoretical model was developed based on mass transfer in a small capillary. The model allows to calculate the driving force as well as the membrane mass and volumetric fluxes for a given set of parameters specifying the membrane pore geometry and the surface properties, such as the water contact angle, pore radius, tortuosity factor, and pore length. Further, to validate the model experimentally, a novel anisotropic super-hydrophobic membrane was fabricated and tested in DCMD mode for desalination of NaCl solution. The DCMD experiments were conducted at various inlet feed temperatures, including a temperature that is very close to the ambient feed. Thus, this work is the first attempt to demonstrate the possibility of designing a MD membrane that is capable of producing water at a small transmembrane temperature difference both theoretically and experimentally.

It should be emphasized that the novel anisotropic membrane could achieve an order of magnitude higher flux than the commercial control membrane, particularly at transmembrane temperature difference as low as 5 °C.

## Experimental

The biporous anisotropic membrane was fabricated by depositing a very thin hydrophobic layer on the hydrophilic porous substrate. The details of the fabrication method are given in Section S1 (S denotes supplementary material).

The morphology and topography (roughness) of the fabricated membrane was characterized using scanning electron microscopy (SEM) and atomic force microscopy (AFM), respectively. Additionally, the wettability of the as-prepared membrane was determined via measuring the water contact angle. X-ray diffraction (XRD) spectra were obtained using Bruker D8 for measuring the d-spacing between adjacent polymer chains in the range of 5Å–80Å at a scanning rate of 0.02Å min^−1^. The d-spacing was calculated using Bragg’s law (nλ = 2d sinθ). The details of each method are given in Section S2.1. The DCMD experiments were conducted by using the DCMD set-up shown in Fig. S1. The details of the experimental procedure are given in Section S2.2.

## Theoretical approaches

**Figure 1:**
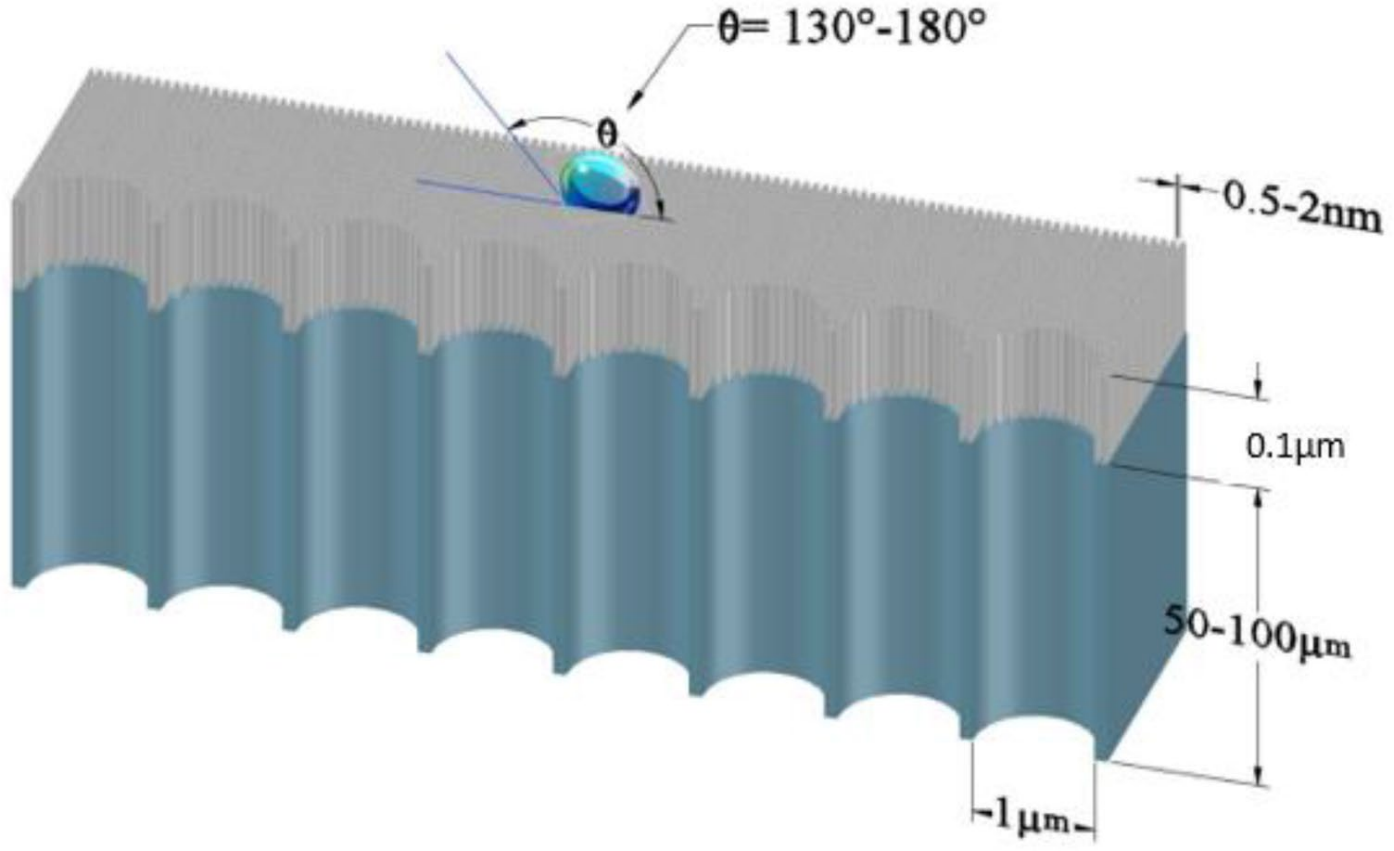
Desired structural characteristics of the active (nano-porous) layer and the support (micro-porous) layer including the range of pore size and thickness for each layer, as well as the active layer hydrophobicity and water contact angle $\theta$ that will achieve a drastic increase in the driving force (vapor pressure difference) with a very small temperature gradient, removing the requirement of large sensible heat supply to the feed solution.

MD is a complex physical process that involves coupled mass and heat transfer phenomena. In this work, the steady-state theoretical model is developed for DCMD by the biporous anisotropic membrane. The upstream side (feed side) of the anisotropic membrane with hydrophobic nano-sized pores, called hereafter “active layer”, is brought into contact with the warm salty feed solution, while the downstream side (permeate side) with pores of much larger sizes, called hereafter “support layer”, is in contact with the colder DI water. Due to the hydrophobicity of the active layer, a water–air interface is formed at the entrance of the nano-sized pores (see Fig. 1), where the saturation vapor pressure is enhanced significantly according to the Kelvin equation (Eq. 1)^14^, which in turn results in a significant improvement of water vapor transport through the membrane^15^.

1 $$ \frac{{p_{s,r} }}{{p_{s} }} = {\text{exp}}\left( { - \frac{{2\sigma V_{m} \cos \theta }}{rRT}} \right) $$

where *p*_*s,r*_ is the vapor pressure in a capillary with a radius *r*, *p*_*s*_ is the vapor pressure at the flat surface, *σ* is the surface tension, *V*_*m*_ is the molar volume of liquid water, *θ* is the contact angle, *R* is the ideal gas constant, and *T* is the absolute temperature. Among those, *p*_*s,*_* σ,* and *V*_*m*_ are a function of temperature, as listed in the supplementary material section S3, Table S1. Equation (1) indicates that when the membrane is hydrophobic, $\theta$ is greater than 90°, leading to $p_{s,r} > p_{s}$. The data presented in Table S2 os Section S3 clearly demonstrate that the capillary effect significantly increases the driving force caused by the increase of vapor pressure at the curved meniscus.

In Table S2, when the feed water of 25 °C comes into contact with the active layer with 1 nm pore radius, the vapor pressure in the pore becomes equal to that of 43 °C of the flat meniscus, which is equivalent to a gain of 18 °C (shown in Table S2 as $\Delta T$). Similarly, when the pore radius is reduced to 0.5 nm the $\Delta T$ will become 41 °C. Therefore, the fundamental concept of the capillary effect on the vapor pressure guides us to the design of a membrane having the biporous anisotropic structure, which we have patented earlier^16^, as illustrated in Fig. 1, i.e. a thin active layer with a large number of nano- or subnano-meter pores, is supported by a thick layer with much larger, possibly in micro-meter range, pores. It is desirable that the active layer is super-hydrophobic to prevent liquid water from entering the pore and also to form a liquid/gas interface with a meniscus that is large enough to allow a significant increase of vapor pressure. The support layer, on the other hand, provides mechanical strength. It is also desirable to maintain the support layer hydrophilic to draw water into the pore so that we can take advantage of the shorter vapor path length and the fast liquid transport via viscous flow, as discussed more in detail in Section S4. Hence, the mass transport is primarily controlled by the vapor transport through the active layer. The membrane, so designed, can significantly reduce the energy consumption in MD, since the heating of feed solution can be minimized, aided by the capillary action of the nano-sized pores.

Regarding the vapor transport across the active layer, the vapor flux (*J*_*W*_) is proportional to the vapor pressure difference (the driving force), as shown in Eq. (2)^17^.

2 $$ J_{w} = B_{m} \left( {p_{f,m} - p_{p,m} } \right) $$

where *B*_*m*_ is the membrane mass transfer coefficient, *p*_*f,m*_ and *p*_*p,m*_ are the vapor pressure at the pore entrance and exit of the active layer, respectively. Herein, the heat transfer resistance at the feed and permeate boundary (including the heat transfer through the support layer due to the high thermal conductivity of the support material) is ignored. This assumption is made to simplify the model equation and, especially, to demonstrate the capillary effect on the vapor transport more clearly. According to the above assumption, $p_{f,m}$ is assumed to be at the feed temperature, while $p_{p,m}$ at the permeate temperature.

It can also be easily assumed that the mass transfer through *nano-sized* pores of the active layer takes place via the Knudsen flow mechanism.

Then,

3 $$ B_{m} = \frac{2}{3}\frac{{\varepsilon_{a} r_{a} }}{{\tau_{a} \delta_{a} }}\left( {\frac{8M}{{\pi RT}}} \right)^{1/2} $$

where $\varepsilon , r, \tau$, and $\delta$ are porosity, radius, tortuosity, and pore length, respectively, and subscript *a* is for the active layer, and $M$ is the molecular weight of water. *T* is the temperature in the pore and the average of feed and permeate temperature is used.

Then,

4 $$ J_{w} = \frac{2}{3}\frac{{\varepsilon_{a} r_{a} }}{{\tau_{a} \delta_{a} }}\left( {\frac{8M}{{\pi RT}}} \right)^{1/2} \times \left( {p_{s,1} \exp \left( { - \frac{{2\sigma_{1} V_{m1} \cos \theta_{1} }}{{r_{a} RT_{1} }}} \right) - p_{s,2} } \right) $$

where subscript 1 and 2 are for feed and permeate, respectively. It should be noted that the capillary effect is ignored at the permeate side of the pore in Eq. (4), which is justified in Section S4.

Furthermore, together with Antoine’s equation,

5 $$ p_{s,i} = exp\left( {23.1964 - \frac{3816.44}{{T_{i} - 46.13}}} \right)\quad i = 1\;{\text{or}}\;2 $$

$J_{w}$ can be obtained as a function of $T_{1}$ for a given set of data on membrane structural parameters, surface tension, contact angle, and permeate temperature.

$J_{w}$ is further normalized with respect to the flux at 25 °C by

6 $$ NJ = \frac{{J_{w,t} }}{{J_{w,25} }} $$

where $J_{w,t}$ and $J_{w,25}$ are $J_{w}$ at temperature *t* (°C) and 25 °C, respectively, to express the effect of temperature for a given $r_{a}$. (Note that in *NJ*, the ramped parameter $\frac{{\varepsilon_{a} r_{a} }}{{\tau_{a} \delta_{a} }}$ is canceled, and *NJ* depends only on *r*_*a*_ and *T*_1_)*.*

## Experimental results

**Figure 2:**
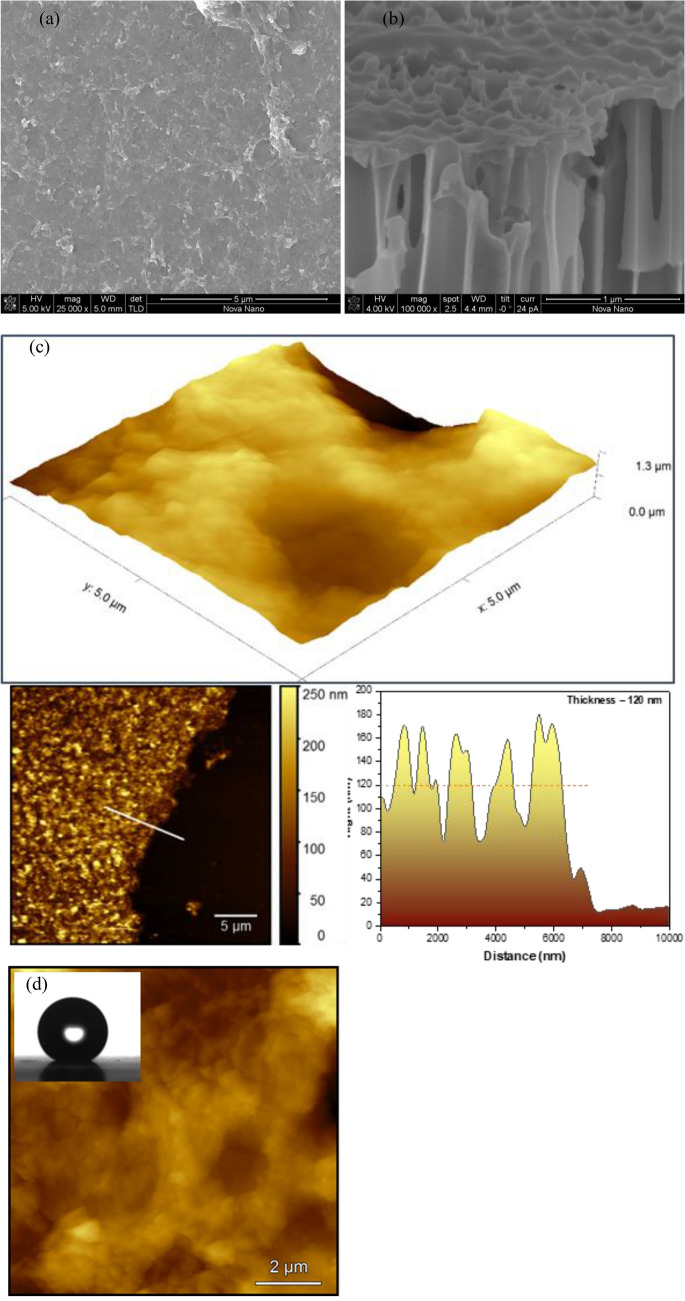
Characterization of the biporous anisotropic membrane (**a**, **b**) surface and cross-sectional SEM images, (**c**) 3D AFM image (5 µm × 5 µm) and thickness profile of active-layer (transferred onto glass substrate), (**d**) 2D AFM image and the inset is for a water droplet on the surface of the active layer, CA = 157.54° ± 11.06°.

Figure 2 summarizes the results of membrane characterization. Figure 2a shows the surface morphology of the biporous anisotropic membrane. In the image, the pores are invisible since they are in the sub-nanometer range. Figure 2b shows the cross-sectional image of the fabricated membrane, in which a very thin active layer seated on an AAO substrate with straight pores is observed. Figure 2c shows the 3D AFM image of the active layer surface and the thickness profile of the active layer (transferred onto a glass slide). The average roughness (Ra) of the synthesized membrane was 131.782 nm, while the average thickness value was 120 nm. Figure 2d showed the 2D AFM images of the membrane, in which no pores of sub-nanometer range could be observed, confirming the SEM results.

Figure 2d (inset) shows the image of a water droplet placed on the surface of the active layer. From the image, a contact angle was measured to be 157.54° ± 11.06° confirming the super hydrophobic property of the active layer surface. On the other hand, the contact angle of the surface of the support layer was 14.85° ± 1.35°, confirming its hydrophilic property. Interestingly, the XRD of the biporous anisotropic membrane showed peaks located at 2θ = 7.3°, 12.3°, and 17°, corresponding to d-spacing of 1.2, 0.7, 0.49 nm (Fig. S2).

**Figure 3:**
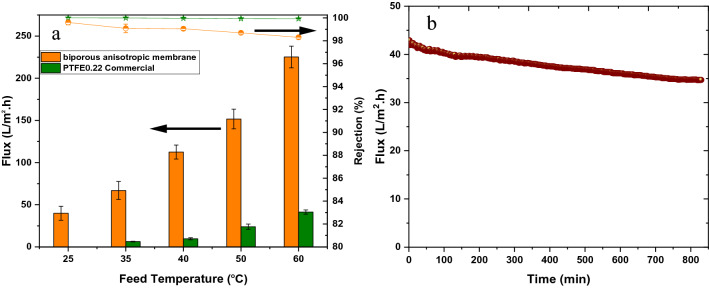
(**a**) Water vapor flux and NaCl rejection of the biporous anisotropic membrane and commercial PTFE membrane. (**b**) Long-term experiment; water flux of the fabricated biporous anisotropic membrane as a function of desalination time using feed at 25 °C. The permeate temperature was kept 20 °C for all experiments, and the flow rate of the feed and permeate was 500 mL/min. The results are obtained using the set-up described in Fig. S1 by the experimental procedure described in Section S2.

Figure 3a shows the results of DCMD experiments using the novel biporous antistropic membrane. The figure also shows the comparison between the novel membrane and a commercial PTFE membrane. As expected, a much higher flux was obtained by the novel biporous membrane. In particular, even at the lowest feed temperature of 25 °C, the biporous membrane exhibited an impressive flux value of 39.9 L/m^2^ h, which increased to 225.2 L/m^2^ h by increasing the feed temperature to 60 °C. These high fluxes were caused mainly by the effect of the capillary force working at the liquid water/gas interface formed at the pore entrance of the active layer, as proven later. As for the salt rejection, it was maintained at nearly 99% or above up to 50 °C but slightly reduced to 98.3% at 60 °C, indicating the occurrence of slight pore wetting. Nevertheless, the fabricated biporous membrane was investigated for long-term desalination to give more meaningful insight for scale-up consideration, and the results are presented in Fig. 3b. As shown in the figure, the water flux slightly decreased from 40 LMH to 34.5 LMH after 14 h operation, indicating good long-term stability of the membrane. However, this flux decrease rate is commonly observed in DCMD operation for closed-loop seawater desalination^18^ and is generally attributed to salt accumulation/deposition on the membrane surface (concentration polarization effect).

It should be noted that a high flux desalination membrane was reported recently by Chen et al.^19^. They have grown a layer of porous carbon structures on a porous ceramic substrate and also achieved high fluxes of about 120 L/m^2^ h (with 3% salt solution at 60 °C) and about 30 L/m^2^ h at 25 °C (Both data taken from Fig. 2b of their work). It should, however, be noted that their experiments were performed using vacuum MD (VMD) with an extra driving force applied on the permeate side. As well, Chen et al.^20^ developed membranes of sub-nanometer pores by co-assembly of graphene oxide nanosheet and polymer on a ceramic substrate and achieved about 100 L/m^2^ h (with 3.5% salt solution at 60 °C) and about 25 L/m^2^ h at feed temperature of 20 °C (Both data taken from Fig. 2d of their work). They have also applied vacuum on the permeate side and called the process pervaporation. Their interpretation of the water transport is, therefore, the fast liquid transport in the capillary, followed by the evaporation at the pore exit, which is different to the mechanism proposed by the current authors.

The following discussion is made to prove that the significant flux improvement is indeed due to the capillary effect on the vapor pressure at the active layer pore entrance.

**Figure 4:**
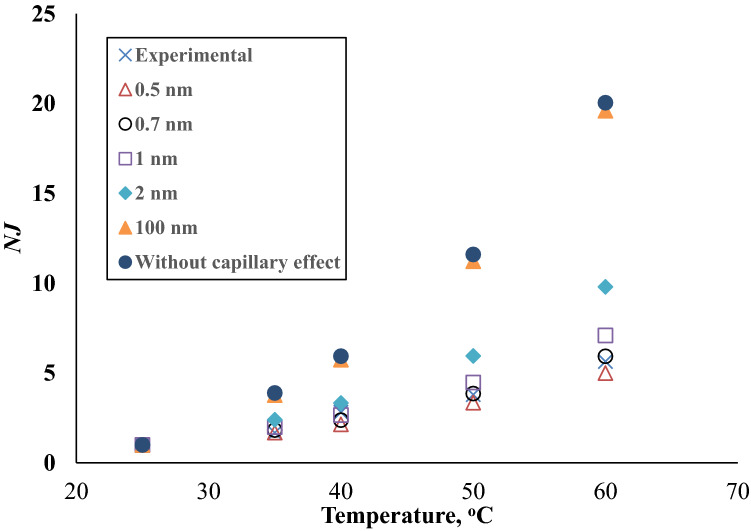
*NJ* (Normalized water vapor flux) versus temperature for different $r_{1}$.

In Fig. 4, the normalized flux *NJ*, is plotted as a function of temperature for different $r_{1} ^{\prime}$ s. The figure shows that *NJ* increases with temperature, and the increase becomes faster as $r_{1}$ increases. Thus, at the highest temperature of 60 °C, *NJ* is 5 and 9.8 for $r_{1} = 0.5\;{\text{and}}\;2.0$ nm, respectively. When $r_{1}$ is as large as 100 nm, *NJ* becomes 19.6, a value close to 20.05, which is the *NJ* that corresponds to the case when $p_{s,r} = p_{s}$, i.e. there is no capillary effect. Thus, steepness of the curve indicates the degree of the capillary effect.

Interestingly, the *NJ* plot obtained from the experimental $J_{w}$ overlaps the curve for $r_{1} = 0.7\;{\text{nm}}$. From these results, it can be safely concluded that the pore size of the active layer of the biporous membrane is 0.7 nm. Interestingly, it is in the range of d-spacing obtained by XRD.

**Figure 5:**
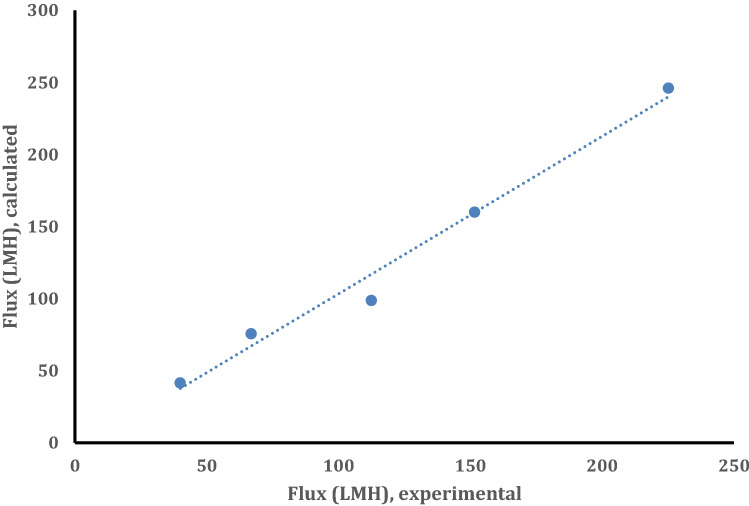
Comparison of calculated and experimental flux based on $\frac{{\varepsilon_{1} }}{{\tau_{{1\delta_{1} }} }} = 6.419 \times 10^{5}$ m^−1^.

Once we know $r_{1}$, we are allowed to calculate the ramped parameter, $\frac{{\varepsilon_{a} }}{{\tau_{{a\delta_{a} }} }}$. For example,$\frac{{\varepsilon_{a} }}{{\tau_{{a\delta_{a} }} }}$ was set equal to 6.419 × 10^5^ m^−1^, and $J_{w}$ was calculated for different temperatures. In Fig. 5, the calculated $J_{w}$ values are correlated to the experimental $J_{w}$. The regression line is slightly off from the origin, having a slope of 1.05. The $R^{2}$ is 0.9825, indicating good agreement between the calculated and experimental values. Using $\delta_{1} = 100\;{\text{nm}}$, the value obtained from the SEM image (Fig. 2b) and assuming $\tau_{1}$ = 1.2, $\varepsilon_{1}$ becomes 0.077. Considering the fractional free volume (FFV), of about 0.05 for a highly crystalline polymer (calculated from the polymer repeat unit of the skin layer using the group contributions^21^ and density of 2 g/cm^3^ for highly fluorinated polymer) the above porosity seems reasonable.

## Conclusions

In summary, we have successfully designed, modeled, and fabricated a novel biporous anisotropic membrane that can be used specifically for the MD process. The key idea is to use the capillary effect to produce a high vapor pressure at the pore inlet, and hence significantly increase the driving force across the MD membrane, enabling the operation of the MD process at low feed temperatures. The proposed biporous anisotropic membrane with the specifically determined characteristics assures a sufficient vapor pressure difference (i.e., the driving force of MD) by the capillary effect, even at ambient feed temperature with a small transmembrane temperature differential (< 5 °C), making MD a highly energy-efficient process, and suitable for large scale module conditions.

Several novel concepts have been successfully proposed to partially overcome the MD obstacles; using surface or joule heating, employing new materials such as carbon nanotubes, etc. However, achieving a promising MD performance at the expense of the energy input or at the high feed temperature is not an ultimate solution, particularly when membranes with a large area are used. Since the overall energy consumption, which is the sum of the heat supply to the feed solution, the conduction heat loss, and the heat loss due to the temperature polarization, increases with the increase in the feed temperature, it is imperative to make the feed temperature as close to permeate temperature as possible. Our theoretical and experimental results exhibited a breakthrough in the MD process. These outstanding results open a promising potential for MD industrialization as a low-cost and high-energy-efficient desalination process, overcoming the main obstacles for MD scale-up.

## Supplementary Information

Supplementary Information.

## Data Availability

The datasets generated during and/or analyzed during the current study are available on reasonable request, by contacting the corresponding author; Mohammed Rasool Qtaishat (m.qtaishat@ju.edu.jo).
