# Supplementary material for: Desalination at ambient temperature and pressure by a novel class of biporous anisotropic membrane
Source: Sci Rep. 2022 Aug 9;12:13564. doi: 10.1038/s41598-022-17876-8 (PMC9363466; doi:10.1038/s41598-022-17876-8)
Supplement: Supplementary file 1 — Supplementary Information. [file 41598_2022_17876_MOESM1_ESM.docx]

**Supplementary Information**

**Desalination at Ambient Temperature and Pressure by a Novel Class of Biporous Anisotropic** **Membrane**

Mohammed Rasool Qtaishat^1,2,3*^, Mohammed Obaid^4^, Takeshi Matsuura^5^, Areej Al-Samhouri^3^, Jung-Gil Lee^6^, Sofiane Soukane^4^, Noreddine Ghaffour^4**^

*^1^Chemical Engineering Department, School of Engineering, The University of Jordan, Amman, 11942, Jordan*

*^2^Arab Open University/ Jordan Branch, Amman, 11731, Jordan*

*^3^Saudi Membrane Distillation Desalination (SMDD) Co. Ltd., King Abdullah University of Science and Technology (KAUST), Innovation and Economic Development, Thuwal 23955-6900, Saudi Arabia*

*^4^King Abdullah University of Science and Technology (KAUST), Water Desalination and Reuse Center (WDRC), Division of Biological & Environmental Science & Engineering (BESE), Thuwal, 23955-6900, Saudi Arabia*

*^5^Chemical and biological Engineering Department, University of Ottawa, 161 Luis Pasteur Street, Ottawa ON. K1N 6N5, Canada*

*^6^Carbon Neutral Technology R&D Department, Korea Institute of Industrial Technology, 89, Yangdaegiro-gil, Seobuk-gu, Cheonan-si, Chungcheongnam-do 31056, South Korea*

**Section S1. Materials and method of membrane preparation**

**Chemicals**

1H,1H,2H,2H-Perfluorododecyltrichlorosilane, (FTCS, 97%, Sigma-Aldrich), m-Phenylenediamine (MPD, Sigma-Aldrich), hexane (98%, Sigma-Aldrich), Anodisc (AAO, a pore size of 0.2 µm, Whatman) were used to prepare the biporous anisotropic membrane without further purification. Commercial Polytetrafluoroethylene (PTFE) MD membrane with a pore size of 0.22 µm was used for the comparison.

**Preparation of the biporous anisotropic membranes**

The biporous anisotropic membrane was fabricated in two steps; firstly, the active layer was synthesized by reacting an aqueous solution containing MPD (MPD/water, 3.0 wt.%) with organic solution containing FTCS (FTCS/Hexane, 0.1 wt.%) at the hexane/water interface. After 2 min, the reaction was stopped by removing the active layer from the interface. Secondly, the active layer was transferred to a water beaker having an AAO support at the bottom to carry the active layer. Finally, the biporous membrane (active layer/AAO support) was cured in an oven at 120^o^C for 3 min.

**Section S2. Characterization of the biporous anisotropic membranes**

**S2.1. Contact angle measurement, Scanning Electron Microscopy (SEM), Atomic Force Microscopy (AFM)**

The water contact angle (WCA) of the biporous anisotropic membrane was measured using CAM 200 goniometer (KSV instruments). A constant volume of DI water droplet (5 μL) was placed on the top surface of the fabricated membrane. The WCA was measured, at least at ten random positions, and the average value was calculated. Scanning Electron Microscopy (SEM, NovaNano 630) was used to characterize the morphology of the fabricated membrane. The membrane sample was coated with 4 nm thick Ir using the Q150T turbo-pumped sputter coater (Quorum Technologies Ltd.), and the SEM images were taken at 5 kV (accelerating voltage) and 56 pA (current) with 5 mm working distance. Atomic Force Microscopy (AFM, Dimension Icon, Bruker, Germany) was used to analyze the surface roughness by acquiring 2D and 3D images at a scan area of 10 µm × 10 µm. The root-mean-square roughness (RMS roughness) was calculated for the fabricated membrane.

**S2.2. MD flux measurement**

The biporous anisotropic membrane was tested in a laboratory-made DCMD set-up (with an effective diameter of 1 cm, Fig. S.1). The flow rate of both feed (30 g/L NaCl) and permeate (DI water) was controlled to 500 mL/min by two pumps (Cole-Parmer). The permeate temperature was maintained at 20 ^o^C by recirculating chilled water for all the experiments, while the feed solutions of various temperatures (25, 35, 40, 50, and 60 ^o^C) were circulated in the feed channel of the module. The vapor flux across the membrane was monitored using a digital balance, placed beneath the permeate beaker, and connected to a PC, whereas the NaCl concentration of the permeate was measured by a conductivity meter. A commercial PTFE membrane with a pore size of 0.22 µm (Membrane Solutions, China), was tested under the same operating conditions as a control membrane.


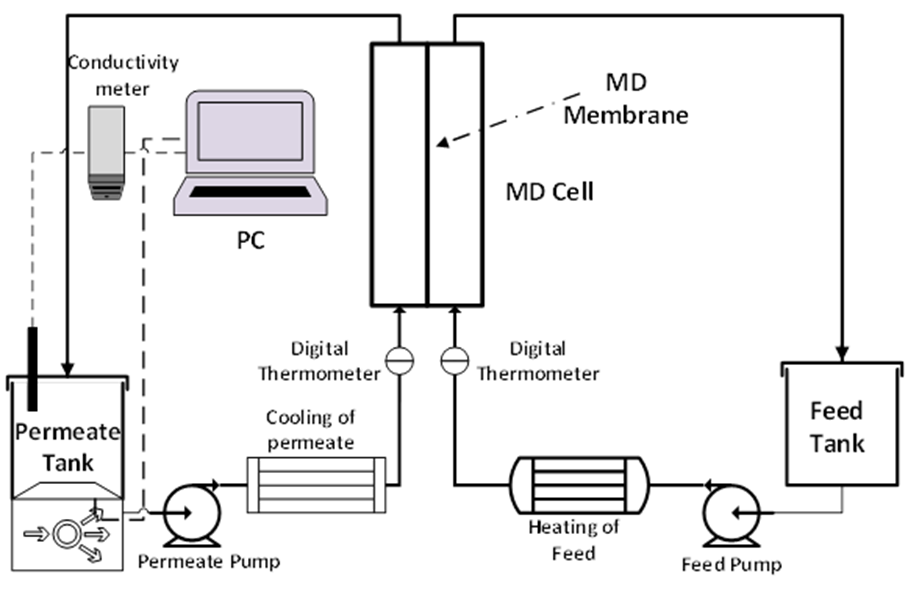


Figure S.1. Schematic of a custom-lab-made DCMD experimental set-up. The digital electronic balance under the permeate tank was used to record the weight gain of the permeate with 1 min interval time to calculate the water vapor flux. The permeate temperature was kept constant at 20 ^o^C using a recirculating chiller, while the feed was heated and stirred using a hot plate stirrer.

**S2.3. XRD results**

XRD results are shown in Fig. S.2

Fig. S.2. XRD results of the biporous anisotropic membrane and the ceramic substrate.

**Section S3. Physical properties of water and the gain in driving force**

The effect of the temperature on the physical properties of water and the gain in driving force by the capillary effect are shown in Table S.1. and Table S.2.

Table S.1. Physical properties of water at different temperatures.

| **Temperature, ^o^C** | **25** °C | **40** °C | **60** °C | **80** °C |
| --- | --- | --- | --- | --- |
| Surface tension (σ, N/m) | 7.20 x 10^-2^ | 6.96 x 10^-2^ | 6.62 x 10^-2^ | 6.26 x 10^-2^ |
| Density (*ρ*, kg/m^3^) | 998.2 | 992.2 | 983.2 | 971.8 |
| Molecular weight (*M*, kg/kmol) | 18.02 | 18.02 | 18.02 | 18.02 |
| Gas constant (*R*), J/kmol K | 8.314 x 10^3^ | 8.314 x 10^3^ | 8.314 x 10^3^ | 8.314 x 10^3^ |
| Vapor pressure (*p_s_*, Pa) | 0.0317 x 10^5^ | 0.0728 x 10^5^ | 0.1993 x 10^5^ | 0.4672 x 10^5^ |
| Molar volume (*V_m,_* m^3^/kmol) | 0.01805 | 0.01815 | 0.01833 | 0.01854 |

Table S.2. The gained driving force as a result of the capillary effect at different membrane pore sizes and different feed temperatures.

|  | ***r* = 0.5 nm** | | ***r* = 1 nm** | | ***r* = 2 nm** | |
| --- | --- | --- | --- | --- | --- | --- |
| **T _feed_** | ***p_s,r_* (kPa)** | ***ΔT* (^o^C)** | ***p_s,r_* (Pa)** | ***ΔT* (^o^C)** | ***p_s,r_* (Pa)** | ***ΔT* (^o^C)** |
| 25^o^C | 25.8 | 41.0 | 9045.8 | 18.0 | 5354.9 | 9.12 |
| 40^o^C | 50.7 | 41.4 | 19212.0 | 19.0 | 11826.4 | 9.14 |
| 60^o^C | 115.0 | 43.2 | 47866.7 | 21.0 | 30886.6 | 9.79 |
| 80^o^C | 227.1 | 40.3 | 103002.2 | 21.0 | 69370.5 | 9.72 |

**^a^ *ΔT*** is the difference between the temperature that is required to produce the saturation vapor pressure $p_{s,r}$ at flat surface and the feed solution temperature, i.e. gained temperature values.

**Section S4. Justification of the negligible liquid water transport in the pores of the support layer and the negligible capillary effect at the small pore exit**

Imagine a biporous structure with a small pore of the active (hydrophobic) layer connected to a large pore of the support layer (hydrophilic) in series (Fig. S.3). The temperature of the support layer is maintained at 20 ^o^C. The active pore entrance faces the feed stream, while the exit of the support layer faces the permeate stream.

Now we assume that both feed and permeate streams are at 20 ^o^C. An air/water interface is formed where the small pore meets the large pore, with air trapped in the hydrophobic small pore and the water that fills the large hydrophilic pore. One can imagine that the position of the interface is neither in the small nor the large pore, since if the interface is in the small pore whose WCA is more than 90^o^, the capillary force will push the interface toward the large pore and vice versa. Besides, the WCA of water at this position should be 90^o^, in order to maintain the balance between the air pressure in the small pore, which is atmospheric pressure + saturation vapor pressure of water at 20 ^o^C and the pressure of the permeate stream, which is the same as that of air inside the pore.

Now let us elevate the temperature of the feed to 60 ^o^C, the highest temperature at which the experiments were conducted. The vapor pressure on the feed side is higher than the vapor pressure on the permeate side and the vapor begins to flow from the feed to the permeate side and condenses at the water/air interface, forming a new interface that is concave upward, and the WCA becomes slightly higher than 90^o^, which causes the flow of liquid water toward the permeate by the capillary force.

The WCA that is necessary to carry the condensed water from the interface to the exit of the large pore can be calculated as follows.

The flux of water at 60 ^o^C is known experimentally to be 225.21 L/m^2^ h, which is nearly equal to 6.15 x 10^-2^ kg/m^2^ s.

Since liquid water flows by the Poiseuille flow mechanism in the large pore,

$J_{w,l}=\frac{\varepsilon_{s}}{\tau_{s}\delta_{s}}\times\frac{\rho r_{s}^{2}}{8\eta}\Delta p$ (S.1)

where$\varepsilon,\tau, \delta\mathrm{and} r$are porosity, tortuosity, length and radius of the pore and the subscript s is for the support layer with large pore size, and they are known to be 0.8, 1.2, 50 x 10^-6^ m, and 0.2 x 10^-6^ m, respectively. $\rho$ and $\eta$ are the density and viscosity of water at 20 ^o^C, respectively.

As for $\Delta p$, it is the capillary pressure, $p_{cap}$ caused by the new meniscus created by the condensation of water at the air-water interface minus the atmospheric pressure.

Inserting all numerical values,

$6.15\times{10}^{-2}=\frac{\left( 0.8 \right)\left( 998 \right)\left( 2\times{10}^{-6} \right)^{2}}{\left( 50\times{10}^{-6} \right)}\times\left( p_{cap}-101,325 \right)$

Therefore,$p_{cap}=101,325+\frac{6.15\times{10}^{-2}}{6.39\times{10}^{-5}}=101,325+962=102,287$ Pa

Corresponding WCA in the small pore is,

${\vartheta=cos}^{-1} \left\{ \frac{-\left( 102,287 \right)\left( {0.7\times10}^{-9} \right)}{2\times\left( 7.286\times{10}^{-2} \right)} \right\}={90.03}^{o}$

This means a small amount of condensed water is enough to drive the water through the large pore to the permeate stream. Hence, there is practically no resistance to the flow of water in the large pore and the mass transport is controlled by the transport through the small pore. As well, the nearly equal to 90^o^ WCA justifies the ignorance of the capillary effect at the small pore exit.


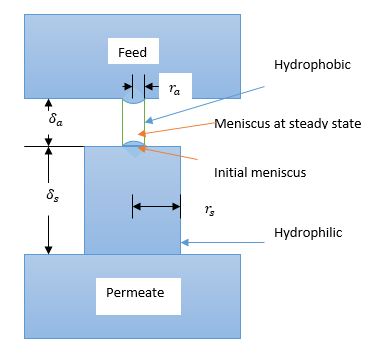


Figure S.3. Schematic illustration of biporous membrane.
